# Amniotic membrane traps and induces apoptosis of inflammatory cells in ocular surface chemical burn

**Published:** 2012-07-26

**Authors:** Ting Liu, Hualei Zhai, Yuanyuan Xu, Yanling Dong, Yajie Sun, Xinjie Zang, Jing Zhao

**Affiliations:** State Key Laboratory Cultivation Base, Shandong Provincial Key Laboratory of Ophthalmology, Shandong Eye Institute, Shandong Academy of Medical Sciences, Qingdao, China

## Abstract

**Purpose:**

Severe chemical burns can cause necrosis of ocular surface tissues following the infiltration of inflammatory cells. It has been shown that amniotic membrane transplantation (AMT) is an effective treatment for severe chemical burns, but the phenotypes of cells that infiltrate the amniotic membrane and the clinical significance of these cellular infiltrations have not previously been reported. The present work studies the inflammation cell traps and apoptosis inducing roles of the amniotic membrane after AMT in patients with acute chemical burns.

**Methods:**

A total of 30 patients with acute alkaline burns were classified as having either moderate or severe burns. In all participants, AMT was performed within one week of his/her injury. After 7–9 days, the transplanted amniotic membranes were removed. Histopathological and immunohistochemical techniques were used for the examination and detection of infiltrating cells, and tests for the expression of CD (cluster of differentiation)15, CD68, CD3, CD20, CD57, CD31, CD147, and CD95 (Fas) were performed. A TUNEL (TdT-mediated dUTP nick end labeling) assay was used to confirm apoptosis of the infiltrating cells. Three patients with herpes simplex-induced keratitis who had undergone AMT to treat persistent epithelium defects were used as a control group. Amniotic membrane before transplantation was used as another control.

**Results:**

After amniotic membrane transplantation, the number of infiltrating cells in patients with severe burns was significantly higher than in patients with moderate burns or in control patients (p<0.05). Among the severe burns patients, CD15 and CD68 were widely expressed in the infiltrating cells, and CD3, CD20, and CD57 were only found in a small number of cells. Occasionally, CD31-positive cells were found in the amniotic membranes. More cells that were CD147, Fas, and TUNEL positive were found in patients with severe burns than in patients with moderate burns or in control patients.

**Conclusions:**

Neutrophils and macrophages were the main cells that had infiltrated into the amniotic membrane during the acute phase of healing from a chemical burns. AMT can trap different inflammatory cells and induce apoptosis of inflammatory cells in acute ocular chemical burns.

## Introduction

Ocular chemical injuries are an ophthalmological emergency and require intensive evaluation and treatment. An ocular chemical burn can be severe and may be particularly challenging to manage. A severe burn may destroy the ocular surface tissue, including the eyelid, the conjunctiva, and the cornea, and it may thereby cause loosening of the epithelium, necrosis and degeneration of the corneal stroma, inflammation, and neovascularization. In many cases, therapeutic strategies for managing ocular burns are effective for controlling disease, and amniotic membrane transplantation (AMT) has proven to be an effective component of acute ocular burn therapy that aids the process of epithelium repair: patients with moderate burns who receive AMT have a significantly faster rate of epithelial healing [1]. AMT can result in a reduction in ocular surface inflammation and the restoration of stem cell functions during the process of healing from chemical burns [2]. The stroma of the transplanted amniotic membrane can even become integrated into the host corneal tissue. This integration is associated with the formation of adhesion structures that anchor and provide stability to the regenerating corneal epithelium, such as desmosomes and hemidesmosomes [3,4]. According to some reports, corneal limbal or mucosal grafts that included amniotic membrane transplantation have had long-term therapeutic effects in treating total limbal stem cell deficiency [5,6].

Data regarding the degree of ocular surface inflammation following amniotic membrane transplantation are seldom reported, however, primarily because it is difficult to obtain ocular tissue from chemical burn patients. Although impression cytology can be used to acquire some information about the evolution of the corneal surface following moderate alkaline burns, it still has some limitations, such as the limited number of cells that are collected [7,8]. In the present study, we investigate the phenotypes of cells that infiltrated the amniotic membrane following AMT in cases of acute alkaline burn and discuss the possible roles of trapping different inflammatory cells in acute chemical burns. Because of the close adherence between the amniotic membrane and the ocular surface, the infiltrated cells and molecules in the amniotic membrane will partially reflect the inflammation status of the ocular surface during the acute phase of a chemical burn.

## Methods

Using protocols approved by the Ethics Committee of the Shandong Eye Institute, Qingdao, China, this study was conducted as a prospective randomized controlled clinical trial for 32 eyes of 30 patients with acute alkaline burns treated at the Qingdao eye hospital between May and December of 2011. The Roper Hall Classification (RHC) system was used to classify the severity of each patient’s injury, and the severity of the disease simultaneously determined according to a new, modified classification system proposed by Dua et al. [9]. This classification system considers both the extent of limbal involvement (in clock hours) and percentage of conjunctival involvement, and it subsequently tabulates an analog scale that can be used to record the clinical status and grade of ocular surface burns. Patients with Grade II and Grade III burns were classified as having "moderate" burns, and patients with Grade IV burns were assigned to a "severe" burns group. Twenty-two eyes of 20 patients were included in the moderate burns group, and 10 eyes of 10 patients were included in the severe burns group. The etiology of the moderate burns group was ammonia in six cases, potassium hydroxide in five cases, lime in seven cases, and sodium hydroxide in two cases. The etiology of the severe burns group was ammonia in two cases, lime in five cases, and sodium hydroxide in three cases. Three patients with Herpes simplex virus keratitis (HSK) were recruited to form a control group; patients in this group received AMT to treat large areas of epithelial defects that had accumulated over a long period of time. Amniotic membrane before transplantation was used as another control.

### Medical treatment

All burns patients were initially treated with conventional medical therapy that included irrigation of sterile saline solution, removal of remaining chemical particles and necrotic tissues, topical administration of topical ofloxacin (0.3%) (Santen, Osaka, Japan) every 6 h, preservative-free tear substitutes every 2 h, topical dexamethasone and homatropine (2%; Alcon, Ft. Worth, TX) twice daily, and orally administered vitamin C every 6 h for four weeks. Eye drops that contained 0.5% timolol maleate or systemic acetazolamide were prescribed for antiglaucoma therapy. HSK patients also received the administration of topical ofloxacin (0.3%) every 6 h and preservative-free tear substitutes every 2 h, and also 0.1 Acyclovir every 6 h.

### Amniotic membrane transplantation

Amniotic membrane samples were obtained from a pathogen-free microorganism donor under sterile conditions. The amniotic membranes were treated with an aseptic agent and cleaned, processed and preserved in the Shandong Province Eye bank. Each amniotic membrane was cut into pieces (3 cm×3 cm) and stored in sterile glycerol. AMT was performed within seven days of chemical burns. Each amniotic membrane was thawed before being transplanted into an eye with ocular burns. The membrane was transferred onto the entire ocular surface with the epithelial side facing up and stromal side of the membrane touching the surface of the eye. Consecutive perilimbal 10–0 Vicryl sutures and interrupted conjunctival sutures were used to anchor the membrane to the underlying conjunctiva and episclera. The amniotic membrane was spread over the entire ocular surface to the fornices. After a period of 7–9 days, the transplanted amniotic membranes were removed for further surgery or other treatment.

### Histopathology and immunohistochemical detection

The amniotic membranes were carefully flattened and half-cut along the central line. The tissues were dehydrated in a series of graded ethanol baths and then in a xylene bath before being immersed in paraffin wax. The samples were then embedded in paraffin molds, sliced into 4-μm thick sections, and mounted on glass slides. To determine whether the central portions of amniotic membranes were representative of the entire membranes, serial 4-μm sections were cut through the entire central amniotic membrane at 200-μm intervals. The slides were found to be consistent from section to section and representative sections from the central portion of the amniotic membranes selected for further evaluation. Hematoxylin and eosin staining was used for microscopic examination and evaluation. Six high-power fields were randomly chosen from each slide, and the number of inflammatory cells per high-power field counted within each amniotic membrane sample. The slides were all evaluated (blind) for diagnosis by the same pathologist, and the specimens coded on their posterior surfaces. Measurements of the inflammatory cells in each amniotic membrane were made and the means of the measured values calculated.

Immunohistochemical detection was used to investigate the expression of neutrophil marker (cluster of differentiation 15 [CD15]), macrophage marker (CD68), T-cell marker (CD3), B-cell marker (CD20), natural killer cell marker (CD57), endothelial cell marker (CD31), extracellular matrix metalloproteinase inducer (CD147), and a factor related to apoptosis (CD95 [Fas]) in infiltrating cells. For all of the immunohistochemical procedures (which were repeated at least three times for each group), 4-μm sections were obtained from the paraffin-embedded corneal buttons. The antigens were recovered by microwaving the sections for 15 min in an EDTA (ethylene diamine tetraacetie acid) solution. Endogenous peroxidase activity was quenched by incubating the sections in a 3% solution of hydrogen peroxide for five min. Normal goat serum was used to block nonspecific staining. The sections were incubated in a solution that contained one of several primary antibodies (mouse anti- CD15, CD68, CD3, CD20, CD57, CD31, CD147, or Fas; Maxin, Fujian, China) for 60 min at 37 °C, after which they were incubated with a reinforcing agent (Maxim) for 15 min at 37 °C, and finally incubated with HRP conjugated goat anti-mouse IgG for 30 min at 37 °C. Peroxidase activity was visualized by incubating the sections in a solution of diaminobenzidine (DAB; Maxin). Negative controls were performed in the absence of primary antibodies. Finally, the samples were mounted and examined under a microscope (Olympus BX61; Olympus, Tokyo, Japan).

### TUNEL assay

Free 3′-OH DNA ends were detected in situ by the terminal deoxyribonucleotidyl transferase-mediated (TUNEL) labeling method according to the manufacturer’s instructions using the in situ cell death detection kit, POD (Roche, Mannheim, Germany). Briefly, the sections were incubated with terminal deoxynucleotidyl transferase (TDT) and nucleotide mixture in a reaction buffer, and then incubated with an anti-FITC antibody conjugated with horseradish peroxidase. Peroxidase activity was detected by exposure of the sections to 3-Amino-9-ethylcarbazole (AEC) solution (Maxin), which were finally counterstained with hematoxylin staining. For negative controls, nucleotide mixture was used instead of the TDT enzyme solution. Positive cells stained red per high power field were counted.

### Follow-up

The patients were examined on days 1 and 7 after undergoing the AMT procedure; they were then examined weekly for the first month after surgery and biweekly until three months had elapsed. At each follow-up visit, the patients were carefully examined for complications such as symblepharon and suture-induced granuloma, and any complications observed treated. The visual acuity, transplanted membrane status, ocular surface, area of any corneal epithelial defects, extent of corneal vascularization, and degree of anterior chamber inflammation were assessed at each visit.

### Statistical analysis

Significant differences among the three groups were evaluated using Student–Newman–Keuls one-way ANOVAs conducted with SPSS (Statistical Product and Service Solutions; version 13.0). The mean±standard deviations were derived, with a p-value of less than 0.05 considered statistically significant.

## Results

### Clinical treatment

No infection was observed after surgery, and no postoperative complications occurred during the follow-up period among any of the patients in our study. The corneal epithelium of the patients in the moderate burns group were repaired within one week of undergoing AMT in 11 eyes of 10 patients (11/22), and the corneal epitheliums of the remaining patients in the moderate burns group recovered within two weeks. However, none of the corneal epitheliums of the patients in the severe burns group recovered within one week, and the corneal epithelium of only 2 eyes of 2 patients (2/10) recovered within two weeks. The other patients in the severe burns group required a second AMT procedure, with an epithelium recovery period of 6–8 weeks. With the aid of antiglaucoma medication administered during the follow-up period, the intraocular pressures in the eyes that had undergone AMT remained stable. After three months of follow-up visits, visual acuity had increased in 22 eyes of 20 patients (22/22) in the moderate burns group, but only in 3 eyes of 3 patients (3/10) in the severe burns group.

### Histological evaluation

**Figure 1:**
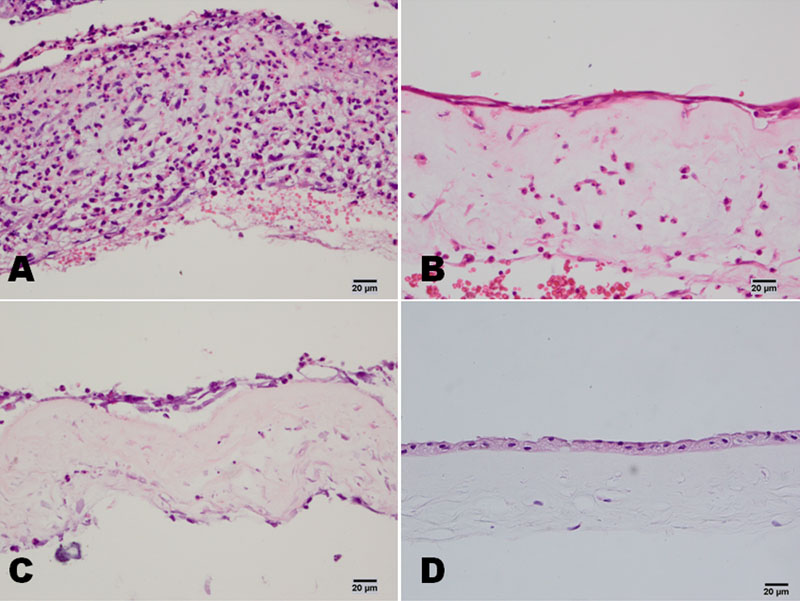
Hematoxylin and eosin staining of ammiotic membrane stromal infiltrating cells in patients with chemical burns. In the severe burns group, there were many neutrophils in the amniotic membrane (**A**). In the moderate burns group, there were clearly fewer neutrophils in the amniotic membrane than in the severe burns group (**B**). In the control HSK group, there were only a few neutrophils on the surface of the amniotic membrane (**C**). In the amniotic membrane before transplantation, no neutrophils were found (**D**). Scale bar: 20 μm.

Many polymorphonuclear leukocytes and mononuclear cells were distributed among the stromas of the amniotic membranes of patients in the severe burns group, and the amniotic membranes were obviously swollen and thick. Compared with patients in the severe burns groups, there were fewer inflammation cells in the amniotic membrane samples taken from patients in the moderate burns group, and their amniotic membranes were less swollen or thick. In the HSK group, there were only a few inflammation-related cells on the surface of the amniotic membrane, and there was no obvious swelling. The histological evaluations of the number of polymorphonuclear leukocytes per high-power field yielded results that reflected significantly larger numbers of cells in samples from patients in the severe burns group than in samples from patients in either the moderate burns or HSK group (p<0.05, for both; Figure 1).

### Immunohistochemistry

**Figure 2:**
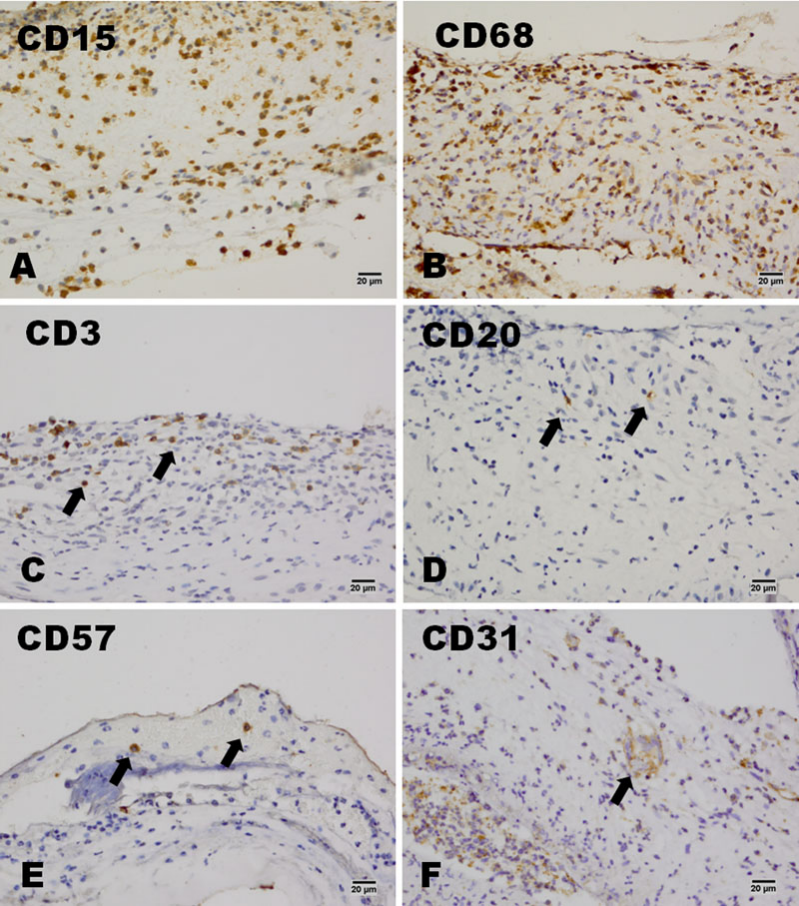
The expression of CD15, CD68, CD3, CD20, CD57, and CD31 in the severe burn group. In the severe burns group, CD15- and CD68-positive cells were the main types of cells that infiltrated the stroma of the amniotic membrane (**A** and **B**). CD3-, CD20-, or CD57-positive cells could also be found in the amniotic membrane (**C**, **D**, and **E**). A few CD31-positive cells could also be found in the amniotic membrane (**F**). Scale bar: 20 μm.

**Figure 3:**
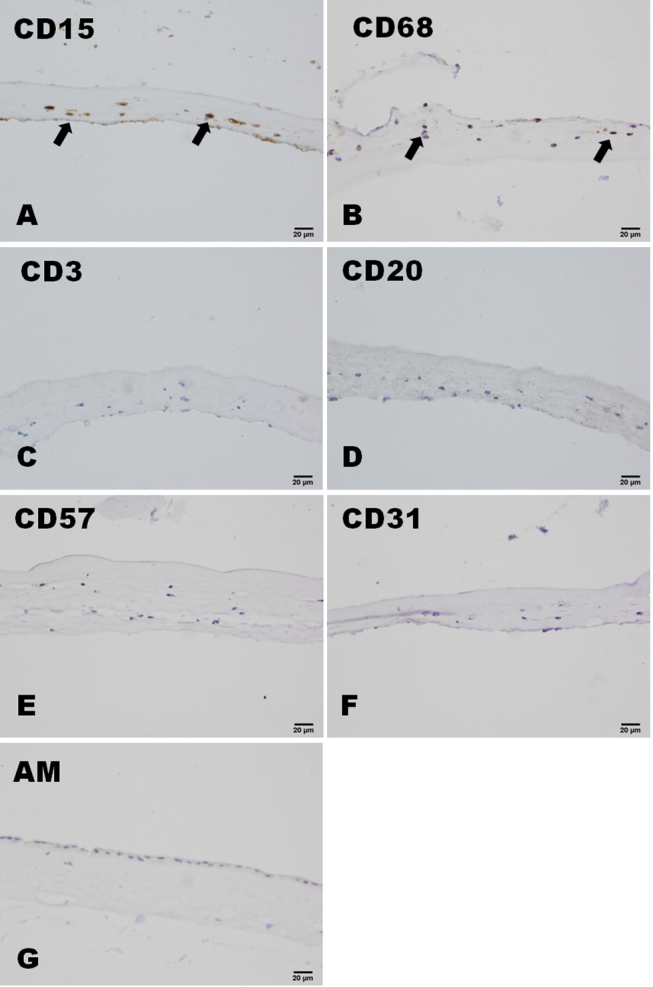
The expression of CD15, CD68, CD3, CD20, CD57, and CD31 in the control HSK group. In the control HSK group, CD15- and CD68-positive cells were the main types of cells that infiltrated the stroma of the amniotic membrane (**A** and **B**). No CD3-, CD20-, CD57- or CD31-positive cells could be found in the amniotic membrane (**C**, **D**, **E**, and **F**). These cell markers could not be found in the amniotic membrane before transplantation (**G**). Scale bar: 20 μm.

**Figure 4:**
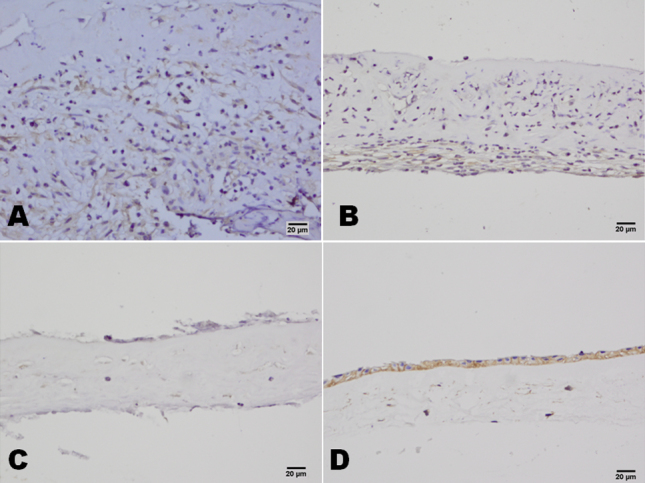
CD147 staining of amniotic membrane stromal infiltrating cells in patients with chemical burns. In the severe burns group, there were many CD147-positive cells in the amniotic membrane (**A**). In the moderate burns group, CD147-positive cells were only observed in the bottom of the amniotic membrane (**B**). In the control HSK group, the cells of the amniotic membrane were CD147-negative (**C**). In the amniotic membrane before transplantation, the epithelium cells of the amniotic membrane were CD147-positive (**D**). Scale bar: 20 μm.

**Figure 5:**
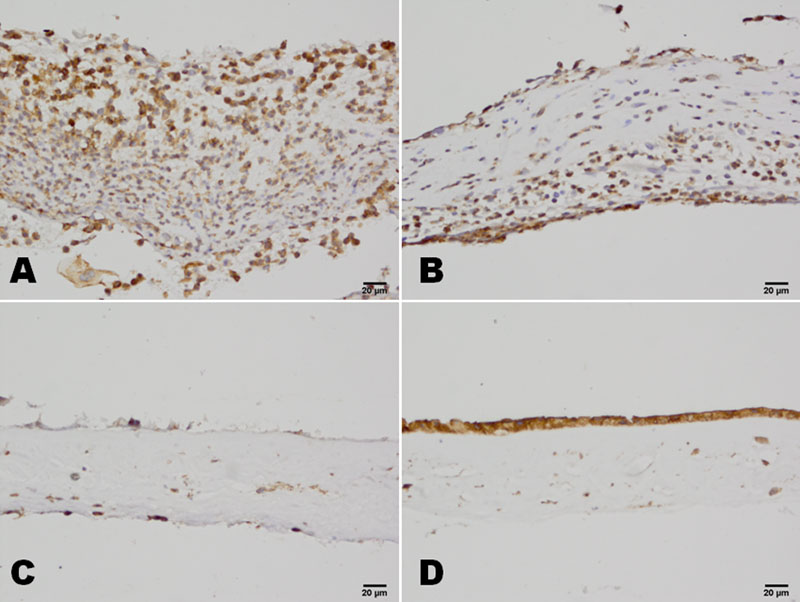
CD95 (Fas) staining of amniotic membrane stromal infiltrating cells in patients with chemical burns. In the severe burns group, there were many Fas-positive cells in the amniotic membrane (**A**). In the moderate burns group, Fas-positive cells were observed in the bottom of the amniotic membrane (**B**). In the control HSK group, a few Fas-positive cells were observed in the surface of the amniotic membrane (**C**). In the amniotic membrane before transplantation, the epithelium cells of the amniotic membrane were Fas-positive (**D**). Scale bar: 20 μm.

In both the severe and the moderate burns groups, CD15 and CD68 were widely expressed in the infiltrated cells, and CD3, CD20, and CD57 found in small numbers of cells. Occasionally, CD31-positive cells were also found in the amniotic membrane samples (Figure 2). In the HSK group, CD15 and CD68 were expressed in the infiltrated cells, and no CD3-, CD20-, CD57-, or CD31-positive cells were found in the amniotic membrane (Figure 3A-F). All these cell markers were negative in the amniotic membrane before transplantation (Figure 3G). In patients in the severe burns group, large numbers of CD147-positive cells were found throughout the amniotic membrane samples, whereas CD147-positive cells were only observed in the bottom of the amniotic membrane samples from patients in the moderate burns group, and patients in the control (HSK) group were CD147-negative. CD147-positive cells were found in the epithelium of amniotic membrane before AMT (Figure 4). More Fas-positive cells were found in the amniotic membrane samples from patients in the severe than the moderate burns group. Fas also strongly expressed in the epithelium cells of amniotic membrane before the AMT, whereas there were only a few Fas-positive cells in the control group (Figure 5).

### TUNEL assay

**Figure 6:**
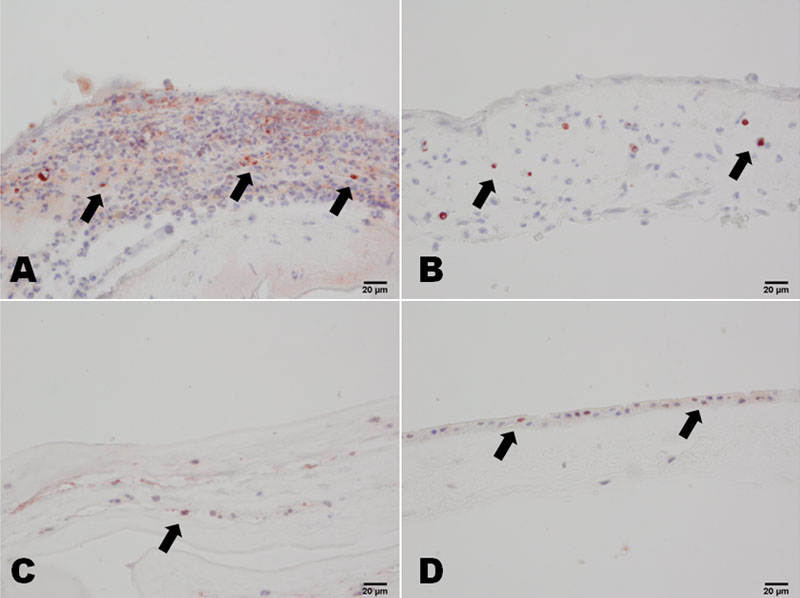
TUNEL staining of amniotic membrane stromal infiltrating cells in patients with chemical burns. In the severe burns group, there were many TUNEL-positive cells in the amniotic membrane (**A**). In the moderate burns group, TUNEL-positive cells were observed in the stroma of the amniotic membrane (**B**). In the control HSK group, a few TUNEL-positive cells were observed in the stroma of the amniotic membrane (**C**). In the amniotic membrane before transplantation, a few epithelium cells of the amniotic membrane were TUNEL-positive (**D**). Scale bar: 20 μm.

In both the severe burns group and the moderate burns group, there were many TUNEL positive cells among the infiltrated cells. TUNEL-positive cells were also found in the amniotic membrane epithelium cells before AMT, whereas there were only a few TUNEL positive cells in the control group (Figure 6).

### Statistic evaluation of amniotic membrane stromal infiltrating cells

**Figure 7:**
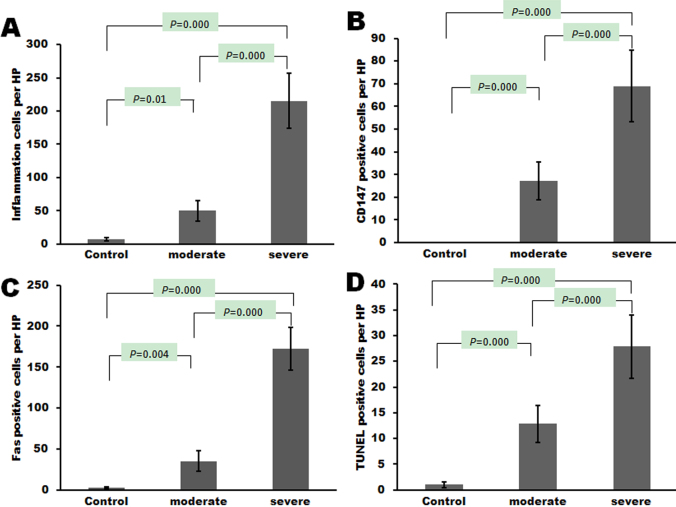
Evaluation of amniotic membrane stromal infiltrating cells in patients with chemical burns. Significant differences were found between the severe burns group and the moderate burns group (p<0.05) in (**A**) the number of inflammation-related cells per high-power field, (**B**) the number of CD147-positive cells per high-power field, (**C**) the number of Fas-positive cells per high-power field, and (**D**) the number of TUNEL-positive cells per high-power field.

Significant differences were found between the severe burns group and the moderate burns group (p<0.05) in the number of inflammation-related cells per high-power field, the number of CD147-positive cells per high-power field, the number of Fas-positive cells per high-power field, and the number of TUNEL-positive cells per high-power field (Figure 7).

## Discussion

At the acute stage of an ocular chemical burn, persistent inflammation prevents epithelialization and accelerates ulceration and melting, with globe perforation. It also contributes to scarring sequela-like symblepharon and lid shortening, tear film deficiency, and inflammatory granuloma in the chronic stage [10,11]. AMT can effectively reduce the degree of inflammation of the cornea and prevent complications such as the development of corneal ulcers and corneal perforation [12-14]. The amniotic membranes provide a mechanical support for ocular surface reconstruction and beneficial biologic properties such as the secretion of cytokines, growth factors, and protease inhibitors. Polymorphonuclear leukocytes (PMNs) are considered to play a central role in the corneal ulceration process that occurs after an alkali burn [15-17]. The infiltration of PMNs and monocytes may occur via the tear film or from the vessels of the corneal limbus. In the present study, CD15 and CD68 were widely expressed in the infiltrated cells. The inflammation cell counts were significantly higher in patients with severe burns than in patients with moderate burns or control patients after AMT. These show that amniotic membrane will trap more inflammation cells during severe chemical burns. At the same time, these cells were induced to expression Fas protein and TUNEL staining positive, which were apoptosis related. Bauer et al. [18] showed that amniotic membrane transplantation induces apoptosis in T lymphocytes in murine corneas with experimental herpetic stromal keratitis. Li et al. [19] also found that amniotic epithelial cells secrete anti-inflammatory and antiproliferative factors that affect the chemotaxis of neutrophils and macrophages and suppress both T- and B-cell proliferation in vitro. We may speculate that the anti-inflammation roles of AMT may not only attract inflammation cells but also induce the apoptosis of these inflammation cells.

CD3, CD20, and CD57 expression were only observed in a small number of cells, which suggests that T cells, B cells and natural killer cells may have limited roles in the immune response during the acute phase of a chemical burn. On occasion, CD31-positive cells were found in the amniotic membrane, which shows that endothelial cells or endothelial progenitor cells migrated to the ocular surface during the acute phase of chemical burns, and may explain the way in which blood vessel formation occurred on the ocular surface. These new blood vessels may allow for the delivery of additional cells and nutrients that assist in ocular surface reconstruction.

High levels of CD147 expression are strongly and independently associated with tumors [20], tissue remodeling [21] and corneal wound healing [22,23]. In the present study, more cells that expressed high levels of CD147 were found in patients with severe burns than in patients with moderate burns or in control patients. This may result from the epithelial-stromal interactions that occur as a result of the disruption of normal anatomic barriers. A study by Gabison et al. [24] showed that CD147 can induce matrix metalloproteinase production and myofibroblast differentiation after direct interaction with corneal fibroblasts. Patients in the moderate burns group have fewer cells that express CD147, which may partially explain the shorter epithelial recovery times of patients in this group relative to that of patients in the severe burns group. CD147-expressing cells were attracted into the amniotic membrane, which resulted in a reduction in MMP production in the corneal stroma, and thereby prevented corneal damage.

AMT alone could not maintain the ocular surface for burns with total limbal ischemia [25,26]. In recent studies, AMT has been shown to provide a surface that is conducive to subsequent ocular surface reconstruction procedures such as auto- and allo-limbal transplantation, and lamellar or penetrating keratoplasty [27,28]. AMT may also be used as a carrier in severe chemical burns cases in which reconstruction of the chemically burned corneal surface requires the use of an ex vivo expanded stem cell allograft or the transplantation of bone marrow-derived human mesenchymal stem cells [29,30]. The amniotic membrane will provide a stem cell carrier as well as an anti-inflammation agent. Amniotic membrane is quite useful for managing moderately severe acute ocular chemical injury by facilitating rapid epithelialization, pain relief, and securing ocular surface integrity [31-33]. In the present study, the rate of epithelial healing in patients with moderate burns was significantly faster in patients who received AMT. In patients with severe burns, AMT appears to have a limited role in promoting epithelial healing because severe burns are associated with extensive limbal ischemia and stem cell deficiency. The limited role of AMT in promoting epithelial healing in patients with severe burns may also result from the presence of more severe ocular surface inflammation or the apoptosis of the epitheliums in these cases. Our clinical studies provide a direct clinical sample based proof, and may help further apply the apoptosis target from bench to bedside.

In conclusion, the work reported here only represents a small clinical study with limited samples: standardization and confirmation of these results may be provided with an animal model and larger scale experiment. To precisely compare the infiltrating cells in the amniotic membranes, flow cytometry to quantify the sub-populations of infiltrated cells may provide more detailed evidence. A PCR expression study also will be helpful to reveal the anti-inflammation molecular mechanisms of the AMT. The analysis of the transplanted amniotic membrane will allow for a method of closely monitoring patients with severe chemical burns. Our further studies will be focused on investigating the molecular mechanisms of amniotic membrane transplantation on the healing processes of acute corneal chemical burns.
